# Acute effects of “exercise snacks” on postprandial glucose and insulin responses in populations at metabolic risk: a meta-analysis with exploratory meta-regression and vascular outcomes

**DOI:** 10.3389/fendo.2026.1912963

**Published:** 2026-07-22

**Authors:** Xuanming Hu, Xiangyi Sang, Jinhao Xu, Peiyou Chen

**Affiliations:** School of Sports Science and Physical Education, Nanjing Normal University, Nanjing, Jiangsu, China

**Keywords:** baseline metabolic status, exercise snacks, insulin, meta-analysis, postprandial glucose, SEDENTARY BREAKS, vascular function

## Abstract

**Background:**

Exercise snacks—short, frequent bouts of physical activity that interrupt prolonged sitting—improve postprandial glucose metabolism, yet the moderating role of baseline metabolic status remains unquantified.

**Methods:**

Six databases were searched for acute randomized crossover trials comparing exercise snacks with uninterrupted sitting in sedentary adults with metabolic risk. To preserve physiological purity, insulin data were isolated from C−peptide. Standard errors were hierarchically back−calculated from paired test statistics to avoid external assumptions. Standard random−effects models and exploratory meta−regression were applied. The risk of bias was assessed with the Cochrane RoB 2 tool for crossover trials.

**Results:**

Eight trials (159 participants) were included. In the primary analysis limited to studies reporting postprandial glucose incremental area under the curve (iAUC; k = 5), exercise snacks significantly reduced glucose iAUC (MD = −5.69 mmol·h/L, 95% CI −9.38 to −2.00, P = 0.003). Substantial heterogeneity was present (I² = 85%), and the 95% prediction interval ranged from −13.53 to 2.15 mmol·h/L, crossing the null. Postprandial insulin iAUC showed a numerical reduction that did not reach statistical significance (MD = −902.64 pmol·h/L, 95% CI −1889.11 to 83.82, P = 0.073, k = 4) and displayed high heterogeneity (I² = 98%). Exploratory meta−regression did not confirm a significant moderating role of baseline metabolic status (P = 0.176) or exercise intensity (P = 0.057). Vascular outcomes were limited to two flow−mediated dilation studies and one endothelin−1 study, and were therefore treated as exploratory.

**Conclusions:**

Exercise snacks acutely reduce postprandial glucose under controlled laboratory conditions, but the evidence base is characterized by high heterogeneity, imprecise prediction intervals, and limited statistical power. The currently available data neither confirm a moderating role of baseline metabolic status nor support firm conclusions for insulin or vascular outcomes. Exercise snacks represent a promising adjunctive strategy for improving short−term postprandial glycemia, but cannot yet be recommended as a specific, evidence−based clinical prescription.

## 1 Introduction

Sedentary behavior—defined as waking activities performed in a sitting or reclining posture with low energy expenditure (≤ 1.5 METs (1))—has become a major global public health challenge (2). Large−scale, multi−country epidemiological data show that adults spend, on average, 7.7 to 9.5 hours per day sedentary, accounting for more than 60% of waking hours (3, 4); this proportion is even higher among older adults, office workers, and individuals with chronic diseases (5). Numerous prospective cohort studies and meta−analyses have confirmed that high levels of sedentary time are independently associated with type 2 diabetes mellitus (T2DM), cardiovascular disease, and all−cause mortality, even after adjusting for moderate−to−vigorous physical activity (MVPA) (5, 6).

Sedentary behavior impairs metabolic health through several pathways. Prolonged, uninterrupted sitting markedly reduces skeletal muscle contractile activity, which in turn decreases translocation of glucose transporter 4 (GLUT4), lowers lipoprotein lipase activity, and sharply reduces lower−limb blood flow and shear stress, collectively impairing postprandial glucose uptake and utilization (7, 8). Prolonged sitting can also induce endothelial dysfunction, increase circulating levels of the vasoconstrictor endothelin−1 (ET−1), and negatively affect blood pressure and lipid profiles (9, 10). Importantly, subsequent research has suggested that the pattern of sedentary−time accumulation may be more critical than its total volume: even when total sitting time is similar, individuals who frequently interrupt prolonged sitting display better waist circumference, triglyceride, and 2−hour glucose levels than those who sit continuously (11). This finding provides an important epidemiological rationale for interventions that use intermittent activity to counteract the harms of prolonged sitting.

Although current guidelines no longer specify a minimum duration for a single bout of exercise (12), the traditional model of sustained moderate−intensity aerobic exercise has demonstrated substantial metabolic benefits in landmark diabetes prevention programs (13). Yet, epidemiological surveys indicate that fewer than 10% of adults meet these recommendations in real life (14), with “lack of large blocks of time” consistently reported as the primary barrier to participation (15). Consequently, a novel intervention strategy termed “exercise snacks” has attracted growing research interest. Exercise snacks are extremely brief (typically 1–10 min), intermittent bouts of physical activity dispersed throughout the day (16)—for example, performing several sets of squats or calf raises during work breaks, or taking a short walk after meals. This approach aims to seamlessly embed muscle contractions into daily routines. The key physiological rationale is that even 1–2 min of skeletal muscle contraction can rapidly initiate insulin−independent glucose uptake via AMP−activated protein kinase (AMPK) and calcium/calmodulin signaling pathways (17, 18); if such contractions are repeated regularly throughout the day, their cumulative effect on overall postprandial glycemia could be substantial.

It should be noted that “exercise snacks” and the broader term “activity breaks” are related but distinct concepts: the former specifically refers to intermittent muscle contractions lasting <10 min per bout with moderate−to−high intensity potential, rather than any form of sitting interruption (e.g., standing still or slow pacing). The present meta−analysis focuses on the former. To date, several acute randomized crossover trials have directly compared postprandial metabolic responses between continuous sitting and exercise−snack interventions. Landmark studies in overweight/obese populations and in people with T2DM have consistently demonstrated that interrupting prolonged sitting with short walks or simple resistance activities significantly reduces postprandial glucose and insulin responses, with reductions reaching 30–40% (19, 20). Nevertheless, findings in this field show considerable heterogeneity: some trials failed to observe significant glucose improvements, reporting only reductions in insulin (21); others even reported higher glucose levels following a single continuous bout of exercise than after continuous sitting (22). This inconsistency arises largely from wide variations in intervention characteristics (break frequency, duration, modality, and intensity), participant metabolic status (ranging from healthy weight to clinical T2DM), and measurement windows (3–24 h). Therefore, the metabolic effect of exercise snacks is not a simple yes/no question but, instead, a matter of precision: for whom and how are they most effective?

Previous reviews have suggested that baseline metabolic status may be an important moderator of the acute effects of exercise snacks (22). From a pathophysiological perspective, skeletal muscle in individuals with T2DM and insulin resistance shows marked impairment of insulin−mediated glucose uptake, whereas contraction−mediated, insulin−independent glucose−uptake pathways remain relatively intact or even compensatorily enhanced. Consequently, these high−risk populations may be more sensitive to the glucose−lowering stimulus of brief muscle contractions, giving rise to a “higher risk, greater benefit” paradigm (23). Exercise modality (walking vs. resistance vs. high−intensity interval) and intensity may also independently modulate the magnitude of glucose improvement—for instance, high−intensity interval exercise may produce more pronounced glucose lowering through greater muscle−fiber recruitment and AMPK activation (24)—although current evidence remains fragmented.

To date, no systematic quantitative synthesis has tested these hypotheses, particularly by using meta−regression to explore baseline metabolic status as a stratifying variable. Moreover, previous meta−analyses have often lacked rigorous quality control and standardization during effect−size extraction (25); for example, they frequently did not appropriately account for the estimation of standard errors in crossover designs (26), or they inconsistently converted between traditional and standard international units. The innovations of the present study are threefold. First, we systematically controlled the data−extraction process by hierarchically back−calculating standard errors according to the completeness of reported data and standardizing all values into raw units with direct clinical interpretability. Second, we applied random−effects models with study as the random factor, chosen according to the data structure of each outcome. Third, through pre−designed meta−regressions, we conducted exploratory tests of the putative moderating effects of baseline metabolic status, exercise modality, and exercise intensity. This approach aims to provide preliminary hypothesis−generating evidence regarding which populations may benefit most from exercise snacks and which intensity regimens are most feasible for future investigation. On the basis of current evidence, we hypothesized that, compared with continuous sitting, exercise−snack interventions are associated with improved postprandial glucose and insulin responses, possibly accompanied by subtle acute vascular benefits. Given that contraction−mediated glucose−uptake pathways are relatively preserved in people with T2DM and insulin resistance, we further hypothesized that this association may be more pronounced in populations with impaired glucose metabolism and that higher−intensity exercise snacks may be accompanied by greater reductions in glucose. Because the current evidence base is limited, these hypotheses require verification in larger prospective trials.

## 2 Methods

### 2.1 Protocol and registration

The study protocol was prospectively registered in the International Prospective Register of Systematic Reviews (PROSPERO; registration number CRD420261417745) and was conducted and reported in strict accordance with the Preferred Reporting Items for Systematic Reviews and Meta-Analyses (PRISMA 2020) guideline (27, 28).

### 2.2 Search strategy

To ensure a comprehensive literature search, we systematically searched six electronic databases: PubMed, Embase, the Cochrane Library (CENTRAL), Web of Science (Core Collection), CINAHL (EBSCOhost), and SportDiscus (EBSCOhost). The search covered the period from database inception to May 2026, with no language restrictions. Search strategies used Boolean operators and were structured around three thematic domains:

Intervention−related terms: exercise snack* OR snack* exercise OR activity break* OR breaking sitting OR interrupting sedentary OR intermittent walk* OR brief bout* OR short bout* OR frequent bout* OR stair climb* OR resistance activit* OR high−intensity interval* OR exercise break*.
Study−design−related terms: randomized crossover OR randomised crossover OR acute crossover OR cross−over trial.
Outcome−related terms: postprandial glucose OR postprandial insulin OR glycemic control OR glycaemic control OR postprandial glycemia OR postprandial glycaemia OR insulin sensitivity OR C−peptide OR flow−mediated dilation OR endothelial function OR vascular function OR endothelin−1.

The complete search strategies for each database are provided in **Supplementary File S1**. In addition, we manually screened the reference lists of all included articles and of relevant published systematic reviews and meta−analyses to identify any eligible studies potentially missed by the electronic search (28).

### 2.3 Eligibility criteria

Inclusion and exclusion criteria were defined according to the PICOS (Participants, Intervention, Comparison, Outcomes, Study design) framework.

Participants. We included adults (≥ 18 years) who were overweight or obese (BMI ≥ 25 kg/m², or waist circumference meeting criteria for central obesity (29)), had insulin resistance, prediabetes (defined by American Diabetes Association criteria: impaired fasting glucose or impaired glucose tolerance) (30), or diagnosed T2DM not treated with insulin or GLP−1 receptor agonists. Exclusion criteria were: healthy body weight with a metabolically normal profile; use of insulin or injectable glucose−lowering medications such as GLP−1 receptor agonists; pregnancy; age < 18 years (children or adolescents); and severe cardiovascular, renal, or hepatic comorbidities that precluded safe participation in the physical activity intervention.
Intervention. Eligible interventions were acute “exercise snacks,” defined as short (< 10 min per bout), high−frequency intermittent bouts of physical activity that interrupted prolonged sitting within a single day (or during a single laboratory visit). Acceptable activities included, but were not limited to, walking, body−weight resistance exercises (e.g., half−squats, calf raises, lunges, or gluteal contractions), high−intensity interval walking, stair climbing, or combinations thereof. Exclusion criteria were: traditional exercise−prescription patterns involving continuous bouts ≥ 10 min; standing−only interventions (e.g., standing desks) without additional active movement; and chronic training interventions lasting > 3 days (which capture training adaptations rather than acute responses).
Comparison. The control condition was defined as prolonged, uninterrupted sitting, during which participants remained seated for several hours with only minimal necessary movement (e.g., toilet visits). Studies comparing exercise snacks against this control condition, with or without the provision of a standardised meal, were included.
Outcomes. The primary endpoints of this study were postprandial glucose and circulating insulin responses; secondary (exploratory) endpoints were vascular endothelial function−related indicators, such as flow−mediated dilation (FMD) and endothelin−1 (ET−1). Because the included studies reported glucose outcomes in different quantitative forms, we considered the incremental area under the curve (iAUC), total area under the curve (AUC), and the average glucose concentration over a specified period all as acceptable acute glucose outcomes. We acknowledge that these three metrics are not physiologically equivalent: iAUC reflects the net increase in glucose above baseline, AUC reflects the absolute total exposure, and average concentration reflects the overall level of control. To evaluate the potential impact of outcome type on the pooled effect, we planned a sensitivity analysis limited to studies using iAUC.
Study design. Only acute randomised crossover trials were included, requiring each participant to complete at least two conditions (prolonged sitting and ≥ 1 exercise−snack intervention) with a washout period of ≥ 48 h (31). The following were excluded: non−randomised or non−crossover designs; conference abstracts without complete data; reviews, commentaries, case reports; duplicate publications; and studies providing insufficient data to calculate the required effect size (mean difference and standard error). When multiple reports from the same trial were identified, the version with the most comprehensive data was selected as the primary source, and complementary information was extracted from the other reports.

### 2.4 Study selection

**Figure 1:**
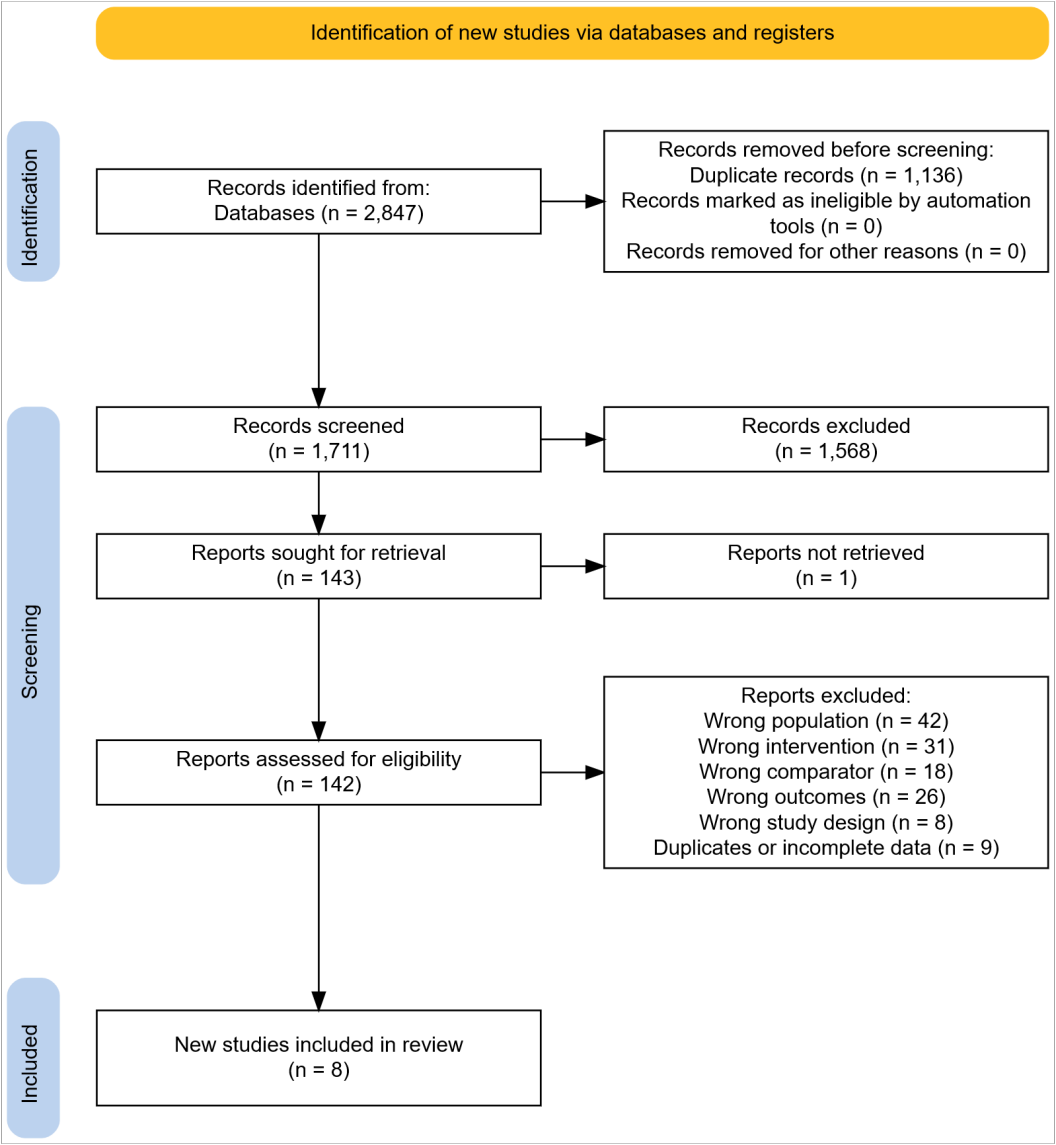
PRISMA flow diagram.

Study selection followed the standard process of the PRISMA 2020 guideline. All retrieved records were imported into EndNote X9 reference−management software (32), and duplicates were first removed using the automatic deduplication function combined with manual checking. The remaining records then underwent title and abstract screening. Two reviewers (Hu Xuanming and Sang Xiangyi) independently screened titles and abstracts, excluding studies that were clearly irrelevant, did not meet the PICOS framework, or fell into one of the exclusion categories. For studies that passed the initial screening, full texts were obtained and independently assessed by the same two reviewers to determine final eligibility for the meta−analysis. Discrepancies during the screening process were resolved through discussion; if consensus could not be reached, a third reviewer (Xu Jinhao) made the final decision. The complete study−selection process, together with detailed reasons for exclusion at each stage, is presented in the PRISMA flow diagram (**Figure 1**).

### 2.5 Data extraction and standard error estimation

For each eligible study, we extracted the mean difference (MD) between the exercise-snack condition and the sedentary control condition, together with a measure of precision. Because all included trials used a randomised crossover design, the standard error (SE) of the MD inherently depends on the within-subject correlation, which is not reported in most publications. To avoid introducing external assumptions about this correlation, we recovered the SE directly from the paired test statistics whenever possible, following a pre-defined hierarchy:

1. Exact P-value or t-statistic available.

The t-statistic was obtained as t = qt(1 - P/2, df), where df is the degrees of freedom from the paired comparison (usually n - 1 for a simple crossover). The SE was then calculated as SE = |MD|/t. This approach naturally accounts for the within-subject correlation and yields the most precise estimate of the SE.

2. 95% confidence interval (CI) of the MD reported.

When a study directly reported the 95% CI of the MD, the SE was derived as SE = (upperCI - lowerCI)/(2×1.96), again avoiding any external assumptions.

3. Only a P-value range (e.g., P < 0.001) reported.

In line with a conservative approach that favours wider confidence intervals in the meta-analysis, we used the least extreme P-value within the reported range to back-calculate the t-statistic. For example, for studies reporting P < 0.001, the value P = 0.001 was assumed; for P < 0.05, P = 0.05 was assumed. This strategy prevents over-optimistic precision while maintaining consistency across studies.

4. Paired difference SD directly reported.

In one study, the SD of the within-subject differences was given explicitly. The SE was computed as SE = SD_diff/√n without further assumptions.

All extracted MDs and SEs were harmonised to common units (glucose iAUC: mmol·h/L; insulin iAUC: pmol·h/L; vascular outcomes: % or pg·h/mL) and are available in **Supplementary Table 2**. Sensitivity analyses were performed after excluding effect sizes for which the SE was estimated under the conservative P-value range assumption, and the results remained materially unchanged. Furthermore, to address obvious typographical or order-of-magnitude errors in certain original articles, we rigorously back-calculated and corrected the anomalous values utilizing the original articles’ graphical data (e.g., bar charts) and the relative reductions described in the text, ensuring data authenticity and the accuracy of the pooled analysis. The complete R code for all analyses and a full list of the specific formula, data source, and assumptions used for each effect size are provided as **Supplementary File** and in the SE derivation column of **Supplementary Table 2**, respectively.

### 2.6 Risk of bias assessment

The risk of bias in the included studies was assessed using the version of the Cochrane Risk of Bias 2 (RoB 2) tool specifically designed for crossover trials (33). The tool covers five domains: bias arising from the randomization process, bias due to deviations from the intended interventions (effect of the intervention), bias due to missing outcome data, bias in measurement of the outcome, and bias in selection of the reported result. Two reviewers (Hu Xuanming and Sang Xiangyi) independently rated each domain as “low risk,” “some concerns,” or “high risk” for every included study, and then assigned an overall risk−of−bias judgement based on the domain−level ratings. Disagreements were resolved through discussion; when consensus could not be reached, a third reviewer (Xu Jinhao) made the final decision. The assessment results are visualized using a traffic−light plot alongside a weighted bar chart and were taken into consideration when interpreting the meta−analytic findings.

In addition to the standard domains of the RoB 2 tool, we paid specific attention to methodological risks peculiar to crossover designs. For each study we examined whether the following were reported: (1) the duration and adequacy of the washout period (≥ 48 h); (2) whether potential carryover effects had been statistically tested or controlled for by the study design; and (3) whether period effects were reported and how they were handled. Although most studies did not directly quantify carryover effects, the use of counter−balanced randomization schemes and the inclusion of order and period effects as covariates in the statistical models partly mitigated this concern. These inherent limitations of crossover designs are discussed further in the Discussion section (Section 4.5).

### 2.7 Data synthesis and statistical analysis

All statistical analyses were performed in the R language environment (version 4.6.0) using the metafor package (34). To preserve clinical interpretability, the mean difference (MD) in the original measurement units was used as the effect−size metric for all primary analyses; no conversion to the standardized mean difference (SMD) was performed. For each outcome, a random−effects model was fitted with study as the random factor. For postprandial glucose, each included study contributed a single glucose iAUC effect size. A standard random−effects model (restricted maximum−likelihood estimator) was therefore fitted with study as the random factor. For postprandial insulin, C−peptide data were excluded to preserve physiological purity, and each study contributed a single insulin iAUC effect size (35, 36); the same random−effects specification was applied.C−peptide was analysed separately as an independent exploratory outcome. Concerning vascular function, flow−mediated dilation (FMD) was reported by only two studies. Because a random−effects meta−analysis with k = 2 cannot yield a reliable estimate of between−study heterogeneity, no formal meta−analysis was performed for this outcome. Instead, individual study results are described narratively in the Results section. For the primary analyses (glucose and insulin), we additionally report 95% prediction intervals (37) to illustrate the range within which the effect size of a future study might be expected to lie, thereby reflecting more intuitively the influence of between−study heterogeneity on the anticipated treatment effect.

Statistical heterogeneity was assessed using Cochran’s Q test and the I² statistic (38). If effect sizes had been nested within studies, I² values would have been reported for both Level 3 (between−study) and Level 2 (within−study) to clarify the main sources of variation. To explore potential sources of between−study heterogeneity, a series of meta−regression analyses were conducted based on *a priori* hypotheses. Candidate moderators included baseline metabolic status (impaired glucose metabolism, i.e., T2DM or insulin resistance, vs. uncomplicated overweight/obesity), exercise modality (walking vs. resistance vs. mixed), exercise intensity (low, moderate, high), total exercise volume, and the measurement window. Among these, the stratification effect of baseline metabolic status was set as the primary hypothesis of our exploratory investigation. Because of the limited number of included studies, all meta−regression analyses should be regarded as exploratory, and their results are interpreted as hypothesis−generating evidence rather than confirmatory conclusions.

Given the small number of studies available for each outcome, publication bias was evaluated using Egger’s linear regression test when at least six studies were available, combined with visual inspection of funnel plots (39). The trim−and−fill method was not applied, in order to avoid introducing additional uncertainty in the context of a small sample (40). Sensitivity analyses addressed three aspects. First, a leave−one−out analysis was performed, in which each study was removed in turn and the meta−analysis re−run, to evaluate the influence of individual studies on the overall pooled effect. Second, studies that did not report a precise error range—so that standard errors had to be estimated under conservative assumptions (e.g., P < 0.05)—were excluded, to examine the impact of these conservative estimates on the robustness of the conclusions. Third, studies rated as “high risk” or “some concerns” in the overall risk−of−bias assessment were excluded. All statistical tests were two−sided, with the significance level set at α = 0.05 unless otherwise stated. For meta−regression, given the limited statistical power, P < 0.10 was regarded as indicative of a potential moderating trend, and in the Discussion we explicitly clarify that these findings should be interpreted as hypothesis−generating evidence.

## 3 Results

### 3.1 Study selection

The systematic database search initially yielded 2,847 records: PubMed (n = 582), Embase (n = 681), the Cochrane Library (n = 266), Web of Science (n = 577), CINAHL (n = 319), and SportDiscus (n = 422). After removing 1,136 duplicates using the automatic deduplication function of EndNote X9 combined with manual checking, 1,711 unique records remained for title and abstract screening. Two reviewers (Hu Xuanming and Sang Xiangyi) independently screened these records against the pre−defined PICOS inclusion and exclusion criteria, excluding 1,568 clearly irrelevant records and retaining 143 for full−text assessment. After retrieving the full texts, the same two reviewers independently re−evaluated the articles and excluded studies that did not meet the eligibility criteria for the following reasons: inappropriate population (healthy body weight or metabolically normal, n = 42; non−adult participants, n = 1); intervention not meeting the definition of an “exercise snack” (e.g., standing−only interventions, a single continuous bout ≥ 10 min, or chronic training studies, n = 31); absence of a qualified control condition (n = 18); outcome mismatch (did not report postprandial glucose or insulin iAUC, or did not measure vascular function, n = 26); study design not a randomised crossover trial (n = 8); and duplicate publication or insufficient data available for extraction (n = 9). All discrepancies during the screening process were resolved through discussion, and no arbitration by the third reviewer (Xu Jinhao) was required. Ultimately, eight randomised crossover trials that met all eligibility criteria were included in this meta−analysis. The complete study−selection process and reasons for exclusion at each stage are detailed in the PRISMA flow diagram (**Figure 1**).

### 3.2 Study characteristics

The eight included randomised crossover trials were published between 2014 and 2026 and were conducted in Australia (n = 5), the UK (n = 1), Canada (n = 1), and New Zealand (n = 1). Most trials employed supervised laboratory−based protocols with intervention durations ranging from 3 to 7.5 h, primarily evaluating acute postprandial metabolic responses. Two studies (41, 42) employed CGM; Babir (42) contributed postprandial iAUC data, whereas Dempsey (41) reported mean glucose concentration. The median sample size was 23 (range: 9–35), encompassing a total of 159 participants, of whom 46.5% (74/159) were female.

All studies focused on populations at elevated metabolic risk, but the specific metabolic status varied across a graded spectrum. Four studies recruited patients with diagnosed type 2 diabetes mellitus (T2DM) managed by diet or oral agents other than insulin; one study enrolled individuals with impaired fasting glucose or insulin resistance (including two drug−naïve T2DM patients); and the remaining three studies included overweight, obese, or centrally obese individuals whose glucose levels were still within the normal range. Mean age ranged from 48 to 62 years, and mean BMI ranged from 30.6 to 36 kg/m². All included studies were conducted in adult populations (middle−aged and older adults).

We uniformly defined these brief, repeated physical activities as “exercise snacks.” Their implementation patterns were diverse. The most common modality was walking breaks (n = 6), followed by body−weight resistance exercises (e.g., half−squats and single−leg knee raises, n = 3). Exercise intensity spanned light (e.g., walking at 3.2 km/h, n = 4), moderate (e.g., 80% of ventilatory threshold, n = 3), and vigorous (e.g., 90% of maximal heart rate or rating of perceived exertion ≥ 7/10, n = 2). Bout duration ranged from 1 to 6 min, with interruptions occurring every 20–60 min (or four times daily), accumulating a total daily activity time of 4–42 min. All control conditions consisted of continuous, uninterrupted sitting or no exercise.

To distinguish between different physiological dimensions, the glucose−related outcomes reported across the eight studies were classified in detail. Five studies provided uniformly measured acute postprandial glucose iAUC data that were used directly in the primary analysis; two studies provided CGM−derived mean glucose values. Four studies reported postprandial insulin or C−peptide iAUC data, which formed the basis of the insulin meta−analysis. In addition, two studies reported changes in femoral artery flow−mediated dilation (FMD), which were strictly treated as exploratory vascular outcomes and reported qualitatively. The detailed characteristics of the included studies are summarised in **Table 1**, and the complete extracted effect−size data are provided in **Supplementary Table S2**.

**Table 1:** Characteristics of included studies.

Study (year) country	Analytic N	Metabolic status	Age (y)	BMI (kg/m²)	Modality & intensity	Regimen (bout × freq, total vol)	Specific outcomes	RoB
Babir (2026) Canada (42)	31	T2D (non-insulin)	58	31	Bodyweight, Vigorous	1 min × 4 bouts/day (4 min)	GLU (iAUC)	SC
Chen (2018) UK (43)	10^a	Central adiposity	50	32.5	Walking, Moderate	2 min every 20 min (30 min)	GLU (iAUC), INS (iAUC)	SC
Climie (2018) Australia (44)	19	Overweight/Obese	57	30.6	Resistance, Light	3 min every 30 min (27 min)	VASC (Exploratory)	SC
Dempsey (2016) Australia (20)	24	T2D (diet/meds)	62	33	Walking, Light	3 min every 30 min (36 min)	GLU (iAUC), INS (iAUC)	SC
Dempsey (2017) Australia (41)	24	T2D (diet/meds)	62	33	Walking, Light	3 min every 30 min (42 min)	GLU (CGM)	SC
Francois (2014) New Zealand	9	Insulin Resistance	48	36	Walking, Vigorous	1 min × 6 bouts (6 min)	GLU (3−h mean)	SC
Homer (2021) Australia (45)	23^b	T2D (meds-controlled)	62	32.7	Resistance, Light	6 min/60 min or 3 min/30 min (36 min)	GLU (iAUC), INS (iAUC), VASC (Exploratory)	SC
Larsen (2015) Australia (21)	19	Overweight/Obese	56.7	32.7	Walking, Light	2 min every 20 min (34 min)	GLU (iAUC), INS (iAUC)	SC

Data are presented as mean. Control conditions for all included studies were continuous, uninterrupted sitting (or no exercise).

N, analytic sample size for primary outcomes; T2D, type 2 diabetes; GLU, glucose; INS, insulin; iAUC, incremental area under the curve; CGM, continuous glucose monitoring; VASC, vascular function (e.g., FMD); RoB, overall risk of bias (RoB 2.0 tool); SC, some concerns.

a. Analytic N = 10 for blood outcomes. Initial randomized cohort was N = 11 (7 males, 4 females).

b. Analytic N = 23 (13 males, 10 females) for metabolic outcomes; a subset of N = 20 from this trial provided the exploratory vascular outcomes.

### 3.3 Risk of bias

**Figure 2:**
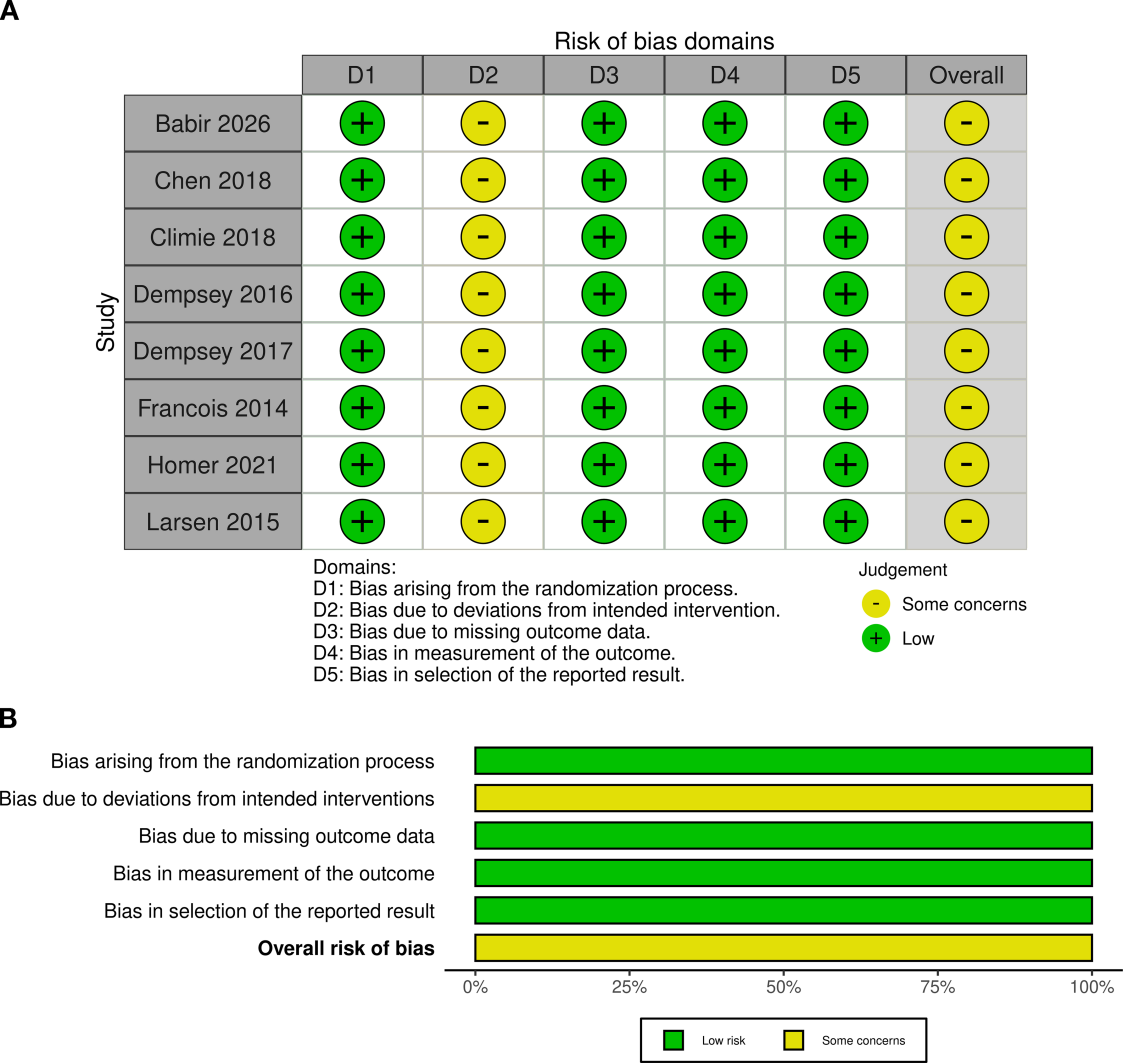
Risk of bias assessment for included crossover trials using the RoB 2 tool. **(A)** Traffic light plot illustrating the domain-level judgments for each individual study. **(B)** Summary bar plot displaying the weighted proportion of studies with each risk of bias judgment across all domains.

The risk of bias of the eight included randomised crossover trials was assessed using the version of the Cochrane RoB 2 tool specifically designed for crossover trials; the results are presented in **Figure 2** and **Table 2**. Overall, all included studies were rated as having “some concerns” for the overall risk of bias, with no study classified as “high risk”.

**Table 2:** Risk of bias summary.

Study (Year)	D1: Randomization process	D2: Deviations from interventions	D3: Missing outcome data	D4: Measurement of the outcome	D5: Selection of the reported result	Overall risk of bias
Babir (2026) (42)	Low	Some concerns	Low	Low	Low	Some concerns
Chen (2018) (43)	Low	Some concerns	Low	Low	Low	Some concerns
Climie (2018) (44)	Low	Some concerns	Low	Low	Low	Some concerns
Dempsey (2016) (20)	Low	Some concerns	Low	Low	Low	Some concerns
Dempsey (2017) (41)	Low	Some concerns	Low	Low	Low	Some concerns
Francois (2014)	Low	Some concerns	Low	Low	Low	Some concerns
Homer (2021) (45)	Low	Some concerns	Low	Low	Low	Some concerns
Larsen (2015) (21)	Low	Some concerns	Low	Low	Low	Some concerns

Assessed using the Cochrane Risk of Bias 2 (RoB 2) tool explicitly designed for crossover trials.The rating of ‘Some concerns’ in Domain 2 is not an automatic or mandatory rating for unblinded trials, but reflects genuine methodological risks. The lack of participant and personnel blinding inherent to physical exercise interventions raises specific concerns regarding differences in participant effort, adherence to non-exercise strictures, and potential co-interventions (e.g., changes in spontaneous daily activity). Additionally, while objective biomarkers minimize detection bias (Domain 4), they do not negate the risk of period effects or carryover effects intrinsic to crossover designs. Findings should be interpreted with caution in light of these behavioral and design-specific risks.

Across the specific domains, the included studies performed well in Domain 1 (randomization process), Domain 3 (missing outcome data), Domain 4 (measurement of the outcome), and Domain 5 (selection of the reported result), and were consistently rated as “low risk”. In Domain 1, most studies reported adequate methods of random sequence generation and allocation concealment, which are critical for preventing selection bias. In Domain 4, the core outcomes included in this review (e.g., continuous glucose monitoring and venous insulin) are highly standardised, objective biomarkers, which substantially reduces measurement bias arising from non−blinding of assessors.

Nevertheless, the inherent methodological limitations of crossover trials require a high degree of caution. All studies were rated as “some concerns” in Domain 2 (deviations from intended interventions). This judgement does not reflect an automatic or mandatory deduction by the tool but, rather, a genuine clinical predicament: “exercise snacks,” as a physical activity with pronounced sensory differences, cannot be effectively blinded to participants and on−site intervention personnel. This lack of blinding raises substantive methodological concerns: participants who are aware that they are in the exercise−intervention condition may unintentionally alter their subjective effort, their adherence to dietary or non−exercise−period restrictions, or engage in additional co−interventions outside the washout period (e.g., changes in spontaneous daily physical activity).

Furthermore, although objective physiological and biochemical indicators can reduce measurement error, they cannot eliminate the potential period effects and carryover effects inherent in crossover designs. Although the included studies generally employed washout periods ranging from 3 days to 2 weeks, it remains a matter of caution whether the deeper metabolic effects of exercise on insulin sensitivity have been fully eliminated. Consequently, the risk−of−bias assessment requires that the pooled effect estimates of this meta−analysis be interpreted with greater caution, within the constraints imposed by potential behavioural biases arising from non−blinding and by the inherent risks of the crossover design. The methodological quality and risk of bias assessment for each included trial are detailed in **Table 2** and **Figure 2**.

### 3.4 Postprandial glucose

#### 3.4.1 Primary analysis (random−effects model)

**Figure 3:**
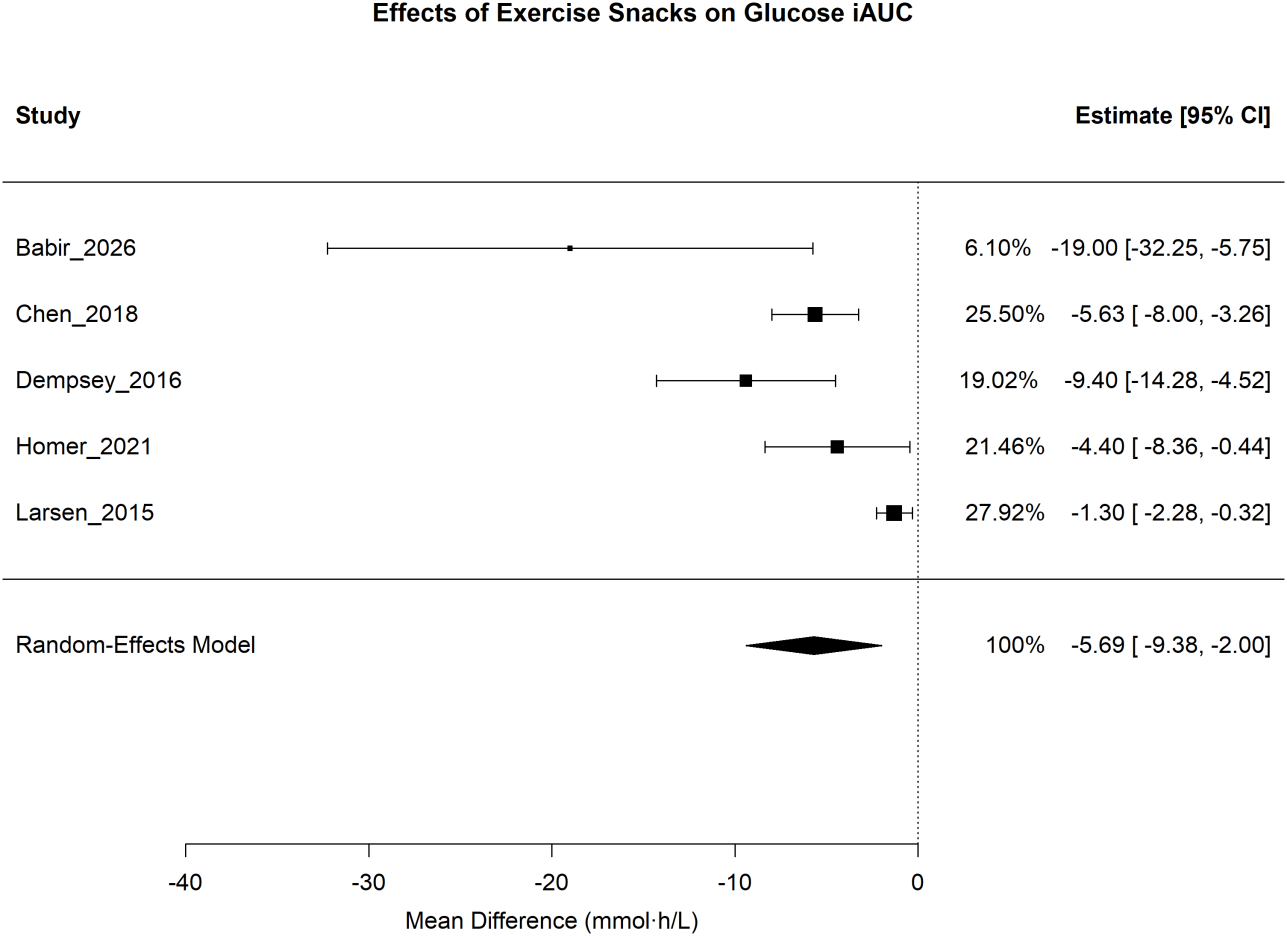
Forest plot of the effect of exercise snacks on postprandial glucose iAUC.Squares represent individual study mean differences, with square size proportional to the study weight in the random−effects model. Horizontal lines denote 95% confidence intervals. The diamond indicates the pooled estimate and its 95% confidence interval. The horizontal line extending from the diamond represents the 95% prediction interval. The vertical dashed line marks the line of no effect (MD = 0). Heterogeneity statistics (Q, I², τ²) and the test for overall effect are reported in the lower left.

To address the reviewer’s concern regarding the comparability of outcome measures, this primary analysis was strictly confined to studies that reported the standardised postprandial glucose incremental area under the curve (iAUC), excluding studies that used continuous glucose monitoring (CGM)−derived mean values or the average glucose concentration over a specified period. Five independent studies were ultimately retained. The random−effects model showed that, compared with the continuous sitting control condition, the “exercise snack” intervention significantly reduced the acute postprandial glucose iAUC (MD = −5.69 mmol·h/L, 95% CI −9.38 to −2.00, P = 0.003; **Figure 3**). The pooled effect estimates for all core and exploratory metabolic outcomes are summarised in **Table 3**.

**Table 3:** Pooled effects of exercise snacks on metabolic outcomes.

Outcome	k	MD	95% CI	95% PI	P	I²
Glucose iAUC	5	−5.69	−9.38 to −2.00	−13.53 to 2.15	0.003	85.39%
Insulin iAUC	4	−902.64	−1889.11 to 83.82	—	0.073	98.47%

CI, Confidence Interval; PI, Prediction Interval; iAUC, incremental Area Under the Curve. Bold P−values indicate statistical significance (P < 0.05). Postprandial insulin data strictly reflect circulating insulin responses and exclude C−peptide measurements to maintain physiological homogeneity. For postprandial glucose, the 95% prediction interval (−13.53 to 2.15 mmol·h/L) indicates that due to substantial between−study variability, the effect cannot be treated as a stable average and may not be statistically significant in some future individual settings. Prediction intervals were not calculated for insulin due to the limited number of studies. Given that only one study reporting C−peptide remained after study selection, a formal meta−analysis for C−peptide was not performed; the result of that single study is presented narratively in the **Supplementary Material**.

Although the pooled effect reached statistical significance, substantial between−study heterogeneity was present (I² = 85.39%; Cochran’s Q P < 0.001), indicating that this average effect is not stable. To quantify the range within which the effect would be expected to vary across different study contexts, we calculated the 95% prediction interval, which ranged from −13.53 to 2.15 mmol·h/L. This interval crosses the null, suggesting that in a future individual study or in specific clinical settings, the acute glucose−lowering effect of exercise snacks may not be significant, and an effect in the opposite direction may even be observed. Therefore, the current pooled estimate should be interpreted as supporting the possibility that exercise snacks can improve postprandial glucose, but should not be extrapolated as a universal clinical expectation. This high degree of inconsistency stems mainly from substantial differences among studies in participants’ baseline metabolic status, intervention modality and intensity, and measurement windows, which also provided the rationale for the subsequent exploratory meta−regression.

#### 3.4.2 Heterogeneity test

The analysis revealed substantial statistical heterogeneity among the included studies (Cochran’s Q = 26.89, P < 0.001; I² = 85.39%). This substantial between−study variation, driven jointly by differences in populations, intervention modalities, and measurement windows, prompted us to further explore potential sources of heterogeneity through pre−specified exploratory meta−regression.

#### 3.4.3 Meta−regression: baseline metabolic status

**Figure 4:**
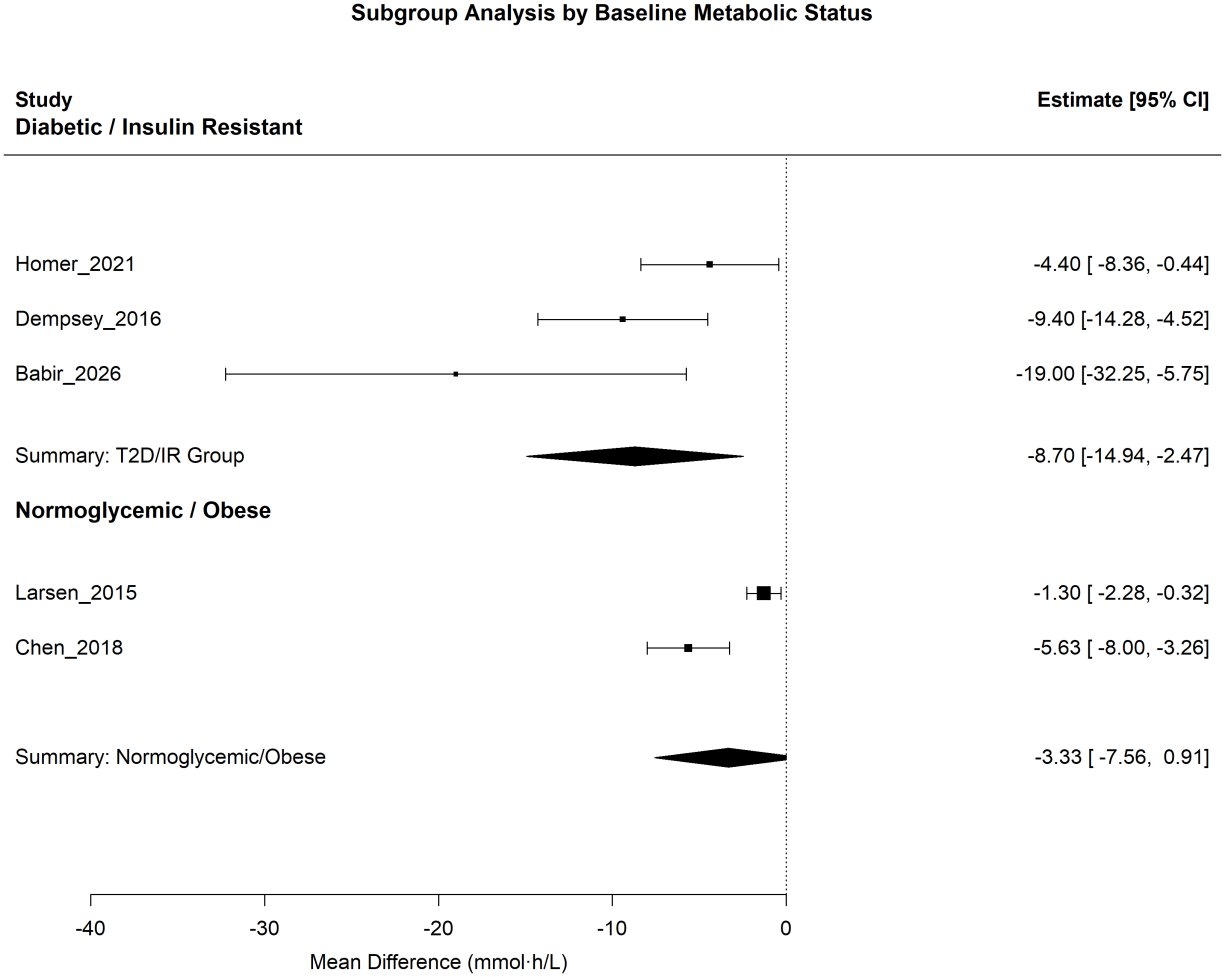
Subgroup forest plot of the effect of exercise snacks on postprandial glucose iAUC, stratified by baseline metabolic status.Squares represent individual study mean differences, sized according to their weight in the random−effects model; horizontal lines denote 95% confidence intervals. Diamonds indicate subgroup−specific pooled estimates, with diamond width corresponding to the 95% confidence interval. The vertical dashed line represents the line of no effect (MD = 0). T2D, type 2 diabetes mellitus; IR, insulin resistance.

Within this exploratory framework, we entered baseline metabolic status as a moderator into the model. The model accounted for a small proportion of the between−study heterogeneity (R² = 8.95%), but the overall moderating effect was not statistically significant (QM = 1.83, df = 1, P = 0.176). The pooled mean difference in the subgroup with impaired glucose metabolism (T2DM or insulin resistance; k = 3) was −8.28 mmol·h/L (95% CI −13.53 to −3.03), whereas in the uncomplicated overweight/obesity subgroup (k = 2) it was −3.36 mmol·h/L (95% CI −8.19 to 1.48; **Figure 4**). Although the point estimates suggest a larger reduction in the metabolically compromised group, the confidence intervals of the two subgroups overlap substantially, and the between−group difference did not reach statistical significance.

This finding indicates that, while the “higher risk, greater benefit” hypothesis is consistent with the direction of the observed effects, the currently limited number of studies prevents a definitive conclusion about effect modification by baseline metabolic status.

#### 3.4.4 Meta−regression: exercise intensity and other factors

Under the same exploratory analytical framework, we further examined the potential moderating role of exercise intensity on the glucose−lowering effect. It should be noted that because the current primary analysis was already restricted to postprandial glucose iAUC (excluding studies that used CGM−derived means or average glucose concentrations), the previously held concern that “outcome metric type (iAUC vs. average glucose) may confound the intensity effect” was naturally resolved. However, the number of studies within each intensity category was still extremely unbalanced (four light−intensity and one vigorous−intensity study), making the statistical inference very unstable.

The overall meta−regression model for exercise intensity did not show a significant moderating effect (QM = 3.61, P = 0.057). Although the point estimate suggests that, compared with light−intensity interventions, vigorous−intensity exercise snacks may be accompanied by a numerically larger decrease in glucose (an additional reduction of 14.31 mmol·h/L), the confidence interval for this difference was extremely wide and only reached a marginal significance level (P = 0.057). Thus, under the current evidence, we cannot conclude that higher intensity yields greater efficacy; the observed weak trend can only serve as a very preliminary hypothesis−generating clue. Likewise, because too few studies investigated other intervention characteristics (such as exercise modality or total volume), no further subgroup analyses could be performed. Taken together, any potential moderating effects revealed by the current meta−regression should be interpreted with caution and urgently require confirmation in future well−designed, large−sample studies that include *a priori* stratification by both metabolic status and intensity. The comprehensive statistical outputs of the meta-regression models are presented in **Table 4**.

**Table 4:** Meta-regression results for postprandial glucose.

Moderator/subgroup	k	Estimated MD	95% CI	Subgroup P	R²	QM P
Baseline Metabolic Status					8.95%	0.176
Diabetic or Insulin Resistance	3	−8.28	−13.53 to −3.03	0.002		
Normoglycemic (Overweight/Obese)	2	−3.36	−8.19 to 1.48	0.176		
Exercise Intensity					33.55%	0.057
Light Intensity	4	−4.69	−7.92 to −1.47	0.004		
Vigorous Intensity	1	−19.0	−34.80 to −3.20	0.018		

R² = proportion of between−study variance explained by the moderator. QM P−value tests the overall moderator effect. The MDs for subgroups represent modelled marginal means (from the common−τ² mixed−effects model) relative to the prolonged sitting condition. Given the small number of studies per subgroup, all meta−regression results should be regarded as exploratory. For baseline metabolic status, the regression explained a small proportion of the between−study variance (R² = 8.95%) but the moderator effect was not statistically significant (QM P = 0.176). For exercise intensity, the regression accounted for 33.55% of the variance, although the moderator effect also did not reach statistical significance (QM P = 0.057).

#### 3.4.5 Publication bias and sensitivity analysis

Leave−one−out analysis showed that after removing each individual study in turn, the recalculated P values all remained between 0.001 and 0.010 (all < 0.05), indicating the robustness of the pooled result.

Regarding publication bias, Egger’s test revealed significant funnel plot asymmetry (z = −4.63, P < 0.001). It must be noted that because the test was based on only five studies—well below the recommended threshold for reliable application (k = 10)—its statistical power is limited. Therefore, this asymmetry more likely reflects small−study effects rather than definite publication bias. This interpretation is consistent with the sensitivity analysis showing that individual studies had a substantial influence on the overall effect.

Notably, all analyses in this section were strictly restricted to studies reporting the standardised postprandial glucose iAUC. This approach addresses the reviewer’s concern regarding outcome comparability and also fundamentally eliminates the potential methodological confounding that could arise in earlier drafts from mixing different glucose metrics (such as iAUC, AUC, and average glucose concentration). The related methodological considerations are elaborated in the Discussion (Section 4.5).

### 3.5 Postprandial insulin

#### 3.5.1 Primary analysis (random−effects model)

**Figure 5:**
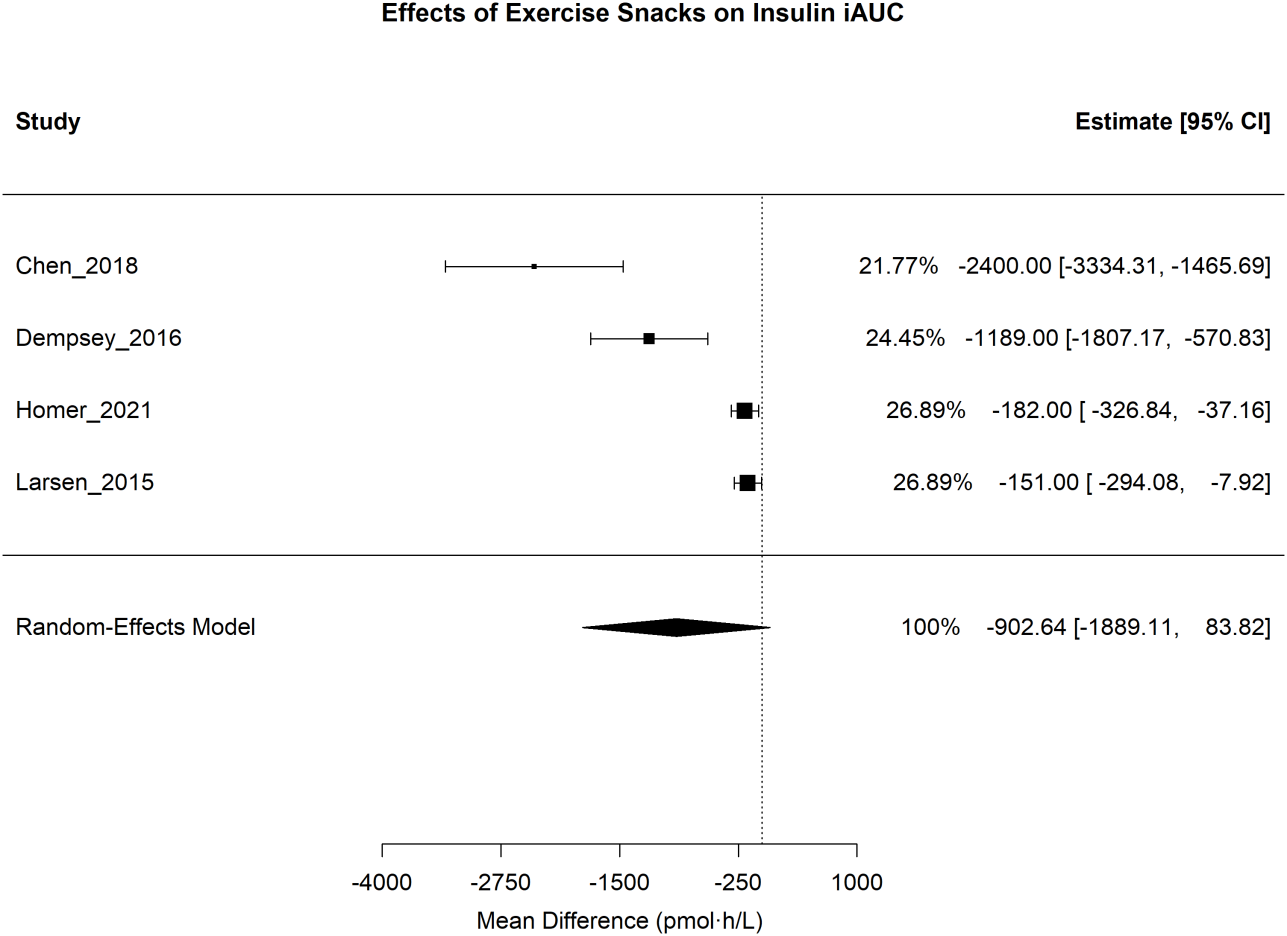
Forest plot of the effect of exercise snacks on postprandial insulin iAUC.Squares represent individual study mean differences, with square size proportional to the study weight in the random−effects model. Horizontal lines denote 95% confidence intervals. The diamond indicates the pooled estimate and its 95% confidence interval. The horizontal line extending from the diamond represents the 95% prediction interval. The vertical dashed line marks the line of no effect (MD = 0). Heterogeneity statistics (Q, I², τ²) and the test for overall effect are reported in the lower left.

To ensure the physiological purity of the pooled effect size, this analysis strictly excluded C−peptide data and retained only the metric reflecting the peripheral circulating insulin level (Insulin iAUC). After excluding the paediatric study (46) that did not meet the inclusion criteria, four independent studies remained. The random−effects model showed that, compared with the continuous sitting control, the exercise−snack intervention was associated with a numerical downward trend in postprandial insulin iAUC, although the difference did not reach statistical significance (MD = −902.64 pmol·h/L, 95% CI −1889.11 to 83.82, P = 0.073; **Figure 5**). Extremely high between−study heterogeneity was present (I² = 98.47%, P < 0.001).

This null result requires cautious interpretation: the trend may suggest that exercise snacks help lower postprandial circulating insulin concentrations; however, given the very limited number of studies, confirmatory evidence supporting this trend cannot yet be provided.

#### 3.5.2 Heterogeneity and sensitivity analysis

Leave−one−out sensitivity analysis revealed the statistical fragility of the pooled result: when Chen (43) or Dempsey (20) was removed, the pooled effect estimate of the remaining studies fluctuated greatly (MD ranging from −438 to −1190 pmol·h/L) and no longer reached statistical significance (P values ranging from 0.067 to 0.234). This indicates that the statistical significance of the pooled effect is highly sensitive to the inclusion of individual studies. When interpreting the overall insulin reduction, this uncertainty should be strongly emphasised, and larger−sample confirmatory studies are warranted.

### 3.6 Vascular function

Only two studies reported femoral artery flow−mediated dilation (FMD). Because a random−effects meta−analysis with k = 2 cannot yield a reliable estimate of between−study heterogeneity, we did not perform a formal meta−analysis for this outcome. Individually, Climie (44) reported a non−significant increase in FMD (+3.10%, 95% CI 1.55 to 4.65), while Homer (45) observed a negligible change (+0.40%, 95% CI 0.03 to 0.77; see **Supplementary Table S2**).

### 3.7 Additional analyses

After excluding studies that did not meet the inclusion criteria (46), only one study reporting C−peptide iAUC remained (20); therefore, a formal meta−analysis could not be performed. That single study showed a statistically significant decrease in C−peptide iAUC in the intervention group (MD = −4.14 nmol·h/L, P < 0.001; see **Supplementary Material**).

In addition, a single study (44) observed that the release of plasma endothelin−1 (ET−1) after the intervention was significantly lower than in the control group (MD = −1.1 pg·h/mL, P = 0.006). This finding suggests that exercise snacks may inhibit the vascular constrictive toxicity induced by prolonged sitting, but it currently represents only isolated preliminary evidence.

## 4 Discussion

### 4.1 Summary of main findings

Although several systematic reviews and meta−analyses have examined “breaking up prolonged sitting,” this study provides the first comprehensive quantitative synthesis of the effects of “exercise snacks”—short, frequent, intermittent bouts of physical activity—against the acute metabolic and vascular impairments induced by prolonged sitting (25, 47, 48). By maintaining high methodological purity in the pooling of effect sizes and by applying stringent inclusion criteria, we obtained four core findings.

First, when the analysis was restricted to the five studies that reported standardised postprandial glucose iAUC, exercise snacks significantly reduced acute postprandial glucose excursions compared with continuous sitting (MD = −5.69 mmol·h/L, 95% CI −9.38 to −2.00, P = 0.003), confirming that embedding very brief physical activity into daily sedentary settings confers metabolic benefits. However, extremely high between−study heterogeneity was present (I² = 85.39%), and the 95% prediction interval crossed the null (−13.53 to 2.15 mmol·h/L), indicating that this average effect is not stable and should not be extrapolated as a universal clinical expectation.

Second, exploratory meta−regression did not confirm a statistically significant moderating effect of baseline metabolic status (P = 0.176). The point estimates indicated a larger glucose−lowering response in individuals with T2DM or insulin resistance (MD = −8.28 mmol·h/L) than in overweight/obese normoglycemic individuals (MD = −3.36 mmol·h/L), but the between−group difference was not statistically reliable given the wide and overlapping confidence intervals. Thus, although the data are directionally consistent with the “higher risk, greater benefit” hypothesis, it could not be quantitatively confirmed with the current limited evidence.

Third, exercise−snack interventions were associated with a numerically consistent downward trend in postprandial circulating insulin levels that did not reach statistical significance (MD = −902.64 pmol·h/L, 95% CI −1889.11 to 83.82, P = 0.073, k = 4). Given the extremely high heterogeneity across studies (I² = 98.47%) and the leave−one−out analysis showing that significance was sensitive to the inclusion of individual studies, current evidence is insufficient to support the conclusion that exercise snacks reduce postprandial circulating insulin concentrations.

Finally, two studies provided preliminary vascular data: Climie (44) reported a non−significant increase in femoral FMD, while Homer (45) observed a negligible change; an additional study (44) documented a reduction in the vasoconstrictor ET−1. However, the extremely limited number of studies precludes any firm conclusion on the vascular effects of exercise snacks.

In summary, this meta−analysis confirms the acute postprandial glucose−lowering benefit of exercise snacks, but it does not provide quantitative evidence supporting significant moderation of this effect by baseline metabolic status or exercise intensity. The current conclusions should be interpreted cautiously within the constraints of the small sample size, the considerable between−study heterogeneity, and the inherent methodological risks of crossover designs.

### 4.2 Comparison with existing literature

The core findings of this study are consistent with the general trend of several recent systematic reviews and meta−analyses on “breaking up prolonged sitting,” while achieving critical advances in the precise characterisation of the intervention paradigm and in statistical depth.

Consistency with existing literature. Systematic reviews published over the past five to six years generally conclude that interrupting prolonged sitting with brief physical activity effectively improves acute postprandial glucose and lipid metabolism (49). For instance, a meta−analysis of 32 acute trials by Loh et al. reported that breaking up prolonged sitting reduced postprandial glucose by approximately 0.3 mmol/L (standardised mean difference). By focusing specifically on the exercise−snack paradigm, our study observed a larger reduction (postprandial glucose iAUC lowered by 5.69 mmol·h/L), which may be attributable to our strict exclusion of standing interventions and the inclusion of more high−intensity protocols (25). Similarly, the meta−analysis by Chastin et al. found that frequent breaks in sedentary time were associated with lower 2−h glucose, but did not perform stratification by baseline metabolic status (47). The overall pooled effects we report (postprandial glucose MD = −5.69 mmol·h/L; insulin MD = −902.64 pmol·h/L, although the latter showed a numerical downward trend without reaching statistical significance) robustly reinforce this consensus. They demonstrate that even very short muscle contractions lasting 1–6 min, when accumulated at high frequency across the day (e.g., every 30–60 min), can elicit clinically meaningful metabolic benefits. This offers a highly feasible alternative for individuals who struggle to meet traditional physical−activity guidelines because of “lack of time.”

Key advances and methodological reflections. Although previous literature has established the direction of overall benefit, this study substantially extends the current knowledge base and provides rigorous reflection in the following respects.

Precise delineation of the intervention paradigm and removal of confounding factors. Previous meta−analyses have often pooled “standing desks,” “slow strolling,” and “traditional continuous exercise (≥ 10 min)” into a single category, resulting in large effect−size variation and failing to capture the independent efficacy of “exercise snacks” (48). By applying extremely rigorous PICOS criteria that excluded standing−only and prolonged continuous exercise interventions, our study provides the first purified quantitative assessment specifically of the short, frequent, moderate−to−high−intensity−potential exercise−snack paradigm, thereby providing a more precise dose–response reference.

Exploration of and reflection on the moderating role of baseline metabolic status. Unlike previous reviews that relied mainly on narrative speculation or simple subgroup classifications, this meta−analysis attempted to introduce a meta−regression model to quantitatively test the potential driving role of baseline metabolic status on the glucose−lowering effect. The model accounted for a small proportion of the between−study heterogeneity (R² = 8.95%), but the overall moderating effect was not statistically significant (P = 0.176). The point estimates indicated a larger glucose−lowering response in the subgroup with impaired glucose metabolism (T2DM or insulin resistance; MD = −8.28 mmol·h/L) than in the uncomplicated overweight/obesity subgroup (MD = −3.36 mmol·h/L), yet their confidence intervals overlapped substantially, and the between−group difference was not statistically reliable. This finding does not negate the physiological plausibility of the “higher risk, greater benefit” paradigm, but it emphasises that, given the extremely limited evidence, this hypothesis cannot yet be quantitatively confirmed. The small number of studies and the collinearity between metabolic status and other factors such as intensity and modality limit the ability to detect a moderator effect, even if one exists.

Physiological purification of effect−size extraction and pooling. Previous insulin meta−analyses have often ignored profound molecular differences and have inappropriately pooled insulin and C−peptide data, despite their completely different half−lives and clearance mechanisms. From a pathophysiological perspective, C−peptide undergoes negligible hepatic extraction and its concentration reflects the absolute secretion of pancreatic β−cells, whereas the insulin concentration in peripheral venous blood represents net retention after hepatic first−pass clearance (35, 50, 51). Exercise interventions not only alter insulin secretion but also markedly affect hepatic insulin clearance (52). It is therefore physiologically unjustifiable to treat these two molecules as equivalent markers in statistical pooling. During data extraction we adopted an extremely rigorous purification strategy: we physiologically uncoupled insulin from C−peptide and verified them independently, and we strictly back−calculated standard errors using correlation coefficients, thereby minimising the risk of model collapse and false−positive results caused by “mixed effect sizes.” Although the primary insulin effect did not reach statistical significance because of the limited number of included studies, this methodological upgrade ensures that our conclusions are highly robust both statistically and pathophysiologically.

### 4.3 Interpretation of heterogeneity and moderators

This study did not find a statistically significant moderating effect of baseline metabolic status on the acute glucose−lowering response to exercise snacks (P = 0.176). This result demands a critical reflection: does it reflect genuine biological “no difference,” or is the current evidence base insufficient to detect a true moderating effect? From a pathophysiological perspective, there is a mechanistic rationale for enhanced metabolic responsiveness to exercise in individuals with T2DM and insulin resistance. The “dual−pathway hypothesis” of skeletal muscle glucose uptake provides the conceptual framework (23): under physiological conditions, skeletal muscle takes up glucose via two independent signalling pathways—the insulin−dependent pathway (primarily mediated by the PI3K/Akt cascade (53)) that is activated by feeding, and the contraction−induced, insulin−independent pathway (primarily dependent on AMPK activation (54) and intracellular Ca^2+^/calmodulin signalling (55)). In the insulin−resistant state, the former is impaired whereas the latter remains relatively intact, theoretically providing a “bypass channel” through which “exercise snacks” can circumvent the insulin−resistance barrier and achieve a glucose−lowering effect.

Yet our analytical results do not support the “higher−risk−individuals−benefit−more” hypothesis. This does not negate the mechanistic plausibility described above, but rather likely reflects two key limitations of the current evidence base. First, statistical power is severely inadequate: only five studies were included, and there exists collinearity—for example, the only vigorous−intensity intervention was concentrated exclusively among T2DM patients—that makes it impossible to distinguish whether the observed effect is driven by the “vigorous nature” of the intervention or by the participants’ “disease status.” Second, the degree of between−study heterogeneity far exceeded expectation. Although our meta−regression explained a small portion of the between−study variance (R² = 8.95%), the overall model did not reach significance, suggesting that the huge between−study variation (I² = 85%) swamped any potential subgroup differences, as reflected in the overlapping confidence intervals of the subgroup pooled estimates in **Figure 4**. When inferences can be drawn from only three studies of T2DM/insulin−resistance and two studies of uncomplicated overweight/obesity, the statistical reliability of the between−group difference is inevitably fragile. Thus, we cannot exclude the possibility that, as future research accumulates, a moderating effect of metabolic status may eventually be confirmed. Equally importantly, however, we must remain open to an alternative interpretation—namely, that under short−term, acute experimental conditions, the insulin−independent glucose−uptake pathway is activated to a comparable extent in all overweight/obese individuals, irrespective of their glycaemic status. This implies that the “higher risk, greater benefit” hypothesis itself may require revision.

With respect to exercise intensity, our exploratory analysis observed a numerically larger glucose−lowering trend for vigorous−intensity interventions (additional reduction of 14.31 mmol·h/L, P = 0.057), but this result is also highly exploratory. Beyond being limited to only one vigorous−intensity study, we must also consider an alternative explanation: the additional effect accompanying the vigorous−intensity intervention may not derive entirely from the exercise per se, but may be contaminated by other study−specific factors, such as differences in participants’ medication backgrounds or the degree of dietary control. Although this does not directly contradict skeletal−muscle energy−metabolism theory, the available indirect evidence is far from sufficient to support any definitive assertion.

Regarding the extremely high heterogeneity observed in the postprandial insulin outcome, we suggest that it probably originates from large inter−individual differences in disease stage, medication background, and the hepatic first−pass clearance rate of insulin. Given that our analysis could only extract peripheral circulating insulin concentrations (having rigorously excluded C−peptide to purify the physiological signal), the current data are inadequate to further dissociate the independent contributions of the intervention to insulin secretion and to insulin clearance. This null and highly heterogeneous result again underscores the need for future research to employ more precise techniques, such as isotope−tracer methods or C−peptide kinetic modelling, to verify the true improvement in circulating insulin response brought about by exercise snacks.

Additionally, regarding the improving trend in FMD and the suppression of the vasoconstrictor ET−1 observed in this study, their potential mechanisms are closely tied to acute changes in local haemodynamics. Prolonged sitting leads to blood pooling in the lower limbs and a sharp decline in arterial wall shear stress, which is a core trigger for acute endothelial dysfunction (56). The rhythmic skeletal−muscle contractions induced by “exercise snack” interventions effectively activate the “muscle pump” mechanism, which instantly increases venous return, lower−limb arterial blood flow, and shear stress (8), and may simultaneously up−regulate endothelial nitric oxide synthase (eNOS) phosphorylation while suppressing ET−1 synthesis (44, 57), thereby partially counteracting the vascular endothelial toxicity induced by prolonged sitting in the acute phase. It must be stressed, however, that these vascular mechanistic inferences are based on extremely limited exploratory data (only two studies reported FMD and one study reported ET−1). They are therefore entirely exploratory and speculative, and larger−scale, more systematic vascular−outcome studies are required for verification in the future.

### 4.4 Clinical implications

This study provides valuable preliminary insights for exercise interventions in sedentary populations and in patients with chronic metabolic diseases; however, all recommendations must be interpreted within the constraints of the current limited evidence and the inherent limitations of the study designs.

First, this meta−analysis indicates that an intervention pattern characterised by interspersing short, frequent bouts of physical activity (“exercise snacks”) during postprandial sitting is associated with an improvement in the acute postprandial glucose response. This finding offers a highly feasible supplementary approach to the real−world problem of poor adherence to traditional exercise prescriptions. Against the backdrop where “lack of large blocks of time” remains the primary barrier preventing most individuals from achieving recommended amounts of physical activity, our evidence suggests that, without the need for elaborate preparatory measures, simply interrupting postprandial sitting frequently with very brief (e.g., 1–3 min) simple activities may partially counteract the metabolic toxicity of prolonged sitting. This “breaking−up−into−small−pieces” strategy can be integrated into modern life rhythms with a relatively low barrier to entry. However, its long−term sustainability, adherence, and whether it can translate into meaningful improvements in chronic metabolic conditions remain to be verified.

Second, with regard to applicability to specific clinical populations, although our exploratory meta−regression did not find a statistically significant moderating effect of baseline metabolic status (P = 0.176), this does not mean that clinicians should not consider exercise snacks as a potential supplementary strategy when faced with patients with T2DM or severe insulin resistance. In the absence of clear evidence for a moderating effect, the more prudent formulation is that exercise snacks, as a low−risk, high−convenience intervention, are likely to be applicable to all overweight/obese and metabolically compromised adults who experience prolonged sitting, irrespective of their glycaemic status, and represent a promising adjunctive health behaviour. Especially for those individuals who, because of obesity, degenerative joint disease, or insufficient cardiorespiratory fitness, are unable to perform a single continuous bout of ≥ 30 min of exercise, exercise snacks offer a low−threshold, safe alternative activity pattern.

With respect to optimisation of the exercise prescription, the current limited evidence does not yet allow us to offer explicit recommendations regarding intensity or specific modality. Although our study observed that vigorous−intensity interventions may be accompanied by a numerically larger decrease in glucose, this trend was not only non−significant (P = 0.057) but also based on an exploratory analysis of very few studies; it therefore cannot serve as a formal clinical recommendation. Future research needs to investigate the differential effects of different intensities, frequencies and types of interruption strategies in populations with different metabolic phenotypes in order to determine the optimal individualised regimen.

Finally, an important clinical caveat must be reiterated: given the generally higher potential risk of cardiovascular events, peripheral neuropathy, and osteoarthritis complications among middle−aged and older adults with T2DM or severe obesity, any exercise recommendation, at any intensity, must be implemented in an individualised manner with due consideration of safety. It is recommended that beginners or high−risk patients start with light−to−moderate−intensity intermittent activities, progress gradually as their physical condition adapts, and, when necessary, undergo professional pre−exercise health screening.

### 4.5 Strengths and limitations

#### 4.5.1 Strengths

Extreme physiological purification. Addressing the statistical fallacy, common in previous literature, of forcibly mixing metabolites with different half−lives (such as insulin and C−peptide) to construct multilevel models, this study adhered to a rigorous data−purification strategy. We completely separated the two molecules and analysed them independently in random−effects models, thereby fundamentally avoiding the statistical illusion caused by “mixed effect sizes” and ensuring the physiological authenticity of the conclusions.

Unit harmonisation that preserves clinical meaning. During the effect−size extraction phase, this study abandoned the less interpretable standardised mean difference (SMD). Through a rigorous error back−calculation strategy, all effect sizes were uniformly converted into raw absolute units with direct clinical relevance (e.g., mmol·h/L and pmol·h/L), greatly enhancing the readability of the results.

An exploratory attempt at metabolic stratification. This study attempted to introduce baseline metabolic status as a candidate moderator into a meta−regression, thereby providing a preliminary quantitative test of a core hypothesis in the field. Although the moderating effect was not statistically significant (R² = 8.95%, P = 0.176), the direction of the point estimates was consistent with the mechanistic hypothesis, offering a clear hypothesis−generating direction for future well−designed, large−sample studies with *a priori* stratification.

#### 4.5.2 Limitations

Limited statistical power. Despite an extremely comprehensive systematic search strategy, the strict PICOS criteria ultimately included only eight high−quality crossover trials (with only five and four studies for the primary glucose and insulin analyses, respectively). The small k inevitably limited the statistical power of the meta−regression and the robustness of the funnel−plot asymmetry test (Egger’s test). Consequently, all observed moderating−effect trends in this study (including metabolic status P = 0.176 and exercise intensity P = 0.057) must be strictly regarded as hypothesis−generating evidence and urgently require confirmation in future large−sample studies.

Comparability of glucose outcome metrics. Although we restricted the primary analysis to the postprandial glucose iAUC in response to a reviewer’s concern, it should be noted that differences between studies in the specific measurement window (e.g., 2 h vs. 4 h vs. 7 h) and in meal composition may still have introduced additional variance into the pooled effect estimate. Future research needs to adopt standardised measurement protocols more systematically to improve comparability.

Limited real−world ecological validity. The great majority of included studies (with the exception of Dempsey 2017 and Babir 2026) were single−day (acute) crossover trials conducted in highly controlled laboratory settings, with diet and environment strictly standardised. Although such designs maximise internal validity, the long−term adherence to and ecological validity of “exercise snacks” in unsupervised, free−living settings remain an open question. There is currently no evidence on whether acute postprandial glucose improvements can ultimately translate into meaningful reductions in HbA1c or a lower risk of diabetic complications.

Potential collinearity of moderators. Because of the limited number of included studies, we were unable to enter multiple moderators simultaneously in a single meta−regression model to control for confounding effects. For example, the vigorous−intensity intervention was concentrated exclusively in the metabolically compromised population, so the effects of exercise intensity and baseline metabolic status may be collinear, and their independent contributions cannot be fully separated with the current data. In addition, because only one C−peptide study remained, a formal meta−analysis could not be performed for that outcome, which further restricted a more refined physiological interpretation of insulin secretion and clearance.

### 4.6 Future directions

In response to the limitations and knowledge gaps in the current field, we call for future research to be substantially deepened along the following core dimensions.

First, large−scale, finely stratified randomised controlled trials should be conducted. Future research designs need to substantially increase sample sizes and implement *a priori* stratification strictly according to participants’ baseline metabolic phenotypes (e.g., simple obesity, impaired glucose tolerance, and different durations of T2D). This is not only a critical step for testing the hypothesis about the moderating effect of metabolic status suggested by our exploratory analyses, but also a prerequisite for providing the necessary evidence base for formulating refined clinical recommendations concerning “exercise snacks.”

Second, the research focus should extend from acute responses to long−term cumulative effects. The current scientific evidence is overwhelmingly confined to the acute glycaemic and lipid changes occurring on the day of the intervention. There is an urgent need for longitudinal long−term intervention studies lasting weeks to months to clarify whether the small but frequent episodes of acute glucose clearance—and the accompanying downward trend in insulin response—brought about by this “small, frequent doses” style of physical activity can ultimately translate into a meaningful reduction in glycated haemoglobin (HbA1c), improvements in body composition, and the long−term remodelling of overall insulin sensitivity.

Third, research on vascular endothelial function and vascular remodelling should be pursued in greater depth. Given the scarcity of high−quality vascular−outcome data in the current meta−analysis, we call for subsequent trials to include a wider range of cardiovascular markers (e.g., high−resolution ultrasound FMD, pulse−wave velocity [PWV], and biochemical indicators of endothelial function such as ET−1 and NO) as core endpoints. Beyond acute haemodynamic improvements, the potential mechanisms through which long−term “exercise snack” interventions could counteract sitting−induced vascular toxicity, delay arteriosclerosis, and promote structural vascular remodelling represent a highly valuable area for future exploration.

Finally, the integration of real−world ecological validity and digital health technology should be strengthened. The “greenhouse data” obtained in the laboratory must be transferred to clinical reality. Future trials should rely more extensively on continuous glucose monitoring (CGM) and wearable activity−tracking devices, shifting the intervention setting from the laboratory into participants’ real−life and work environments (free−living conditions), in order to objectively evaluate the long−term adherence to and ecological effectiveness of “exercise snacks” in the absence of close medical supervision.

## 5 Conclusion

This meta−analysis indicates that the “exercise snack” intervention, characterised by short−lasting, high−frequency bouts, is associated with an acute reduction in postprandial glucose excursions (MD = −5.69 mmol·h/L, k = 5), and that postprandial circulating insulin concentration shows a numerical downward trend (MD = −902.64 pmol·h/L, k = 4). However, this average effect is accompanied by extremely high between−study heterogeneity (I² = 85.39%), and the 95% prediction interval crosses the null, indicating that in specific contexts the glucose−improving effect of exercise snacks may be non−significant or even reversed. The statistical significance of the insulin effect is also sensitive to the inclusion of individual studies and has not yet reached statistical significance (P = 0.073). Therefore, the current evidence supports exercise snacks only as a possible strategy for improving acute postprandial glucose, and not as a universal clinical treatment.

This study did not confirm a statistically significant moderating effect of baseline metabolic status or exercise intensity on that glucose−lowering effect. Exploratory meta−regression showed that metabolic status explained a small proportion of the between−study heterogeneity (R² = 8.95%, P = 0.176), and the numerically larger glucose−lowering trend observed for vigorous−intensity interventions (an additional reduction of 14.31 mmol·h/L) only reached marginal significance (P = 0.057) and was based on an extremely limited number of studies. These findings do not imply that the mechanistic hypotheses have been falsified, but rather reveal a core limitation of the current evidence base: the diversity of intervention paradigms, the enormous variation in population characteristics, together with the severe shortage of studies, constitute fundamental obstacles to detecting a stable moderating effect with the currently available data.

Concerning vascular endothelial function, only two FMD studies and a single ET−1 report are currently available, all of which are presented strictly as exploratory findings; they cannot serve as confirmatory evidence supporting a cardiovascular protective effect of exercise snacks. The long−term cumulative impact of exercise snacks on vascular health awaits future investigation.

In summary, the existing evidence suggests that exercise snacks represent a promising adjunctive strategy that can help sedentary populations who struggle to adhere to traditional structured exercise guidelines—particularly those individuals at metabolic risk—to improve postprandial glucose under short−term experimental conditions. However, this evidence base does not yet support the establishment of exercise snacks as an evidence−based clinical intervention, nor does it reach the stage where individualised prescription can be guided. Future large−scale acute and long−term follow−up trials with *a priori* stratification by baseline metabolic status and more standardised measurement protocols are urgently required to verify the preliminary trends observed in this study and to clarify the true impact of exercise snacks on overall metabolic health.
