# Supplementary material for: Acute effects of “exercise snacks” on postprandial glucose and insulin responses in populations at metabolic risk: a meta-analysis with exploratory meta-regression and vascular outcomes
Source: Front Endocrinol (Lausanne). 2026 Jul 22;17:1912963. doi: 10.3389/fendo.2026.1912963 (PMC13437454; doi:10.3389/fendo.2026.1912963)
Supplement: Supplementary file 1 [file SupplementaryFile1.docx]

The following strategies were run in each database from inception to 15 May 2026. No language restrictions were applied.

PubMed

Search: ("exercise snack*"[tiab] OR "snack* exercise"[tiab] OR "activity break*"[tiab] OR "breaking sitting"[tiab] OR "interrupting sedentary"[tiab] OR "intermittent walk*"[tiab] OR "brief bout*"[tiab] OR "short bout*"[tiab] OR "frequent bout*"[tiab] OR "stair climb*"[tiab] OR "resistance activit*"[tiab] OR "high-intensity interval*"[tiab] OR "exercise break*"[tiab]) AND ("randomized crossover"[tiab] OR "randomised crossover"[tiab] OR "acute crossover"[tiab] OR "cross-over trial"[tiab]) AND ("postprandial glucose"[tiab] OR "postprandial insulin"[tiab] OR "glycemic control"[tiab] OR "glycaemic control"[tiab] OR "postprandial glycemia"[tiab] OR "postprandial glycaemia"[tiab] OR "insulin sensitivity"[tiab] OR "C-peptide"[tiab] OR "flow-mediated dilation"[tiab] OR "endothelial function"[tiab] OR "vascular function"[tiab] OR "endothelin-1"[tiab]).

Embase

Search: ('exercise snack*':ti,ab OR 'snack* exercise':ti,ab OR 'activity break*':ti,ab OR 'breaking sitting':ti,ab OR 'interrupting sedentary':ti,ab OR 'intermittent walk*':ti,ab OR 'brief bout*':ti,ab OR 'short bout*':ti,ab OR 'frequent bout*':ti,ab OR 'stair climb*':ti,ab OR 'resistance activit*':ti,ab OR 'high-intensity interval*':ti,ab OR 'exercise break*':ti,ab) AND ('randomized crossover':ti,ab OR 'randomised crossover':ti,ab OR 'acute crossover':ti,ab OR 'cross-over trial':ti,ab) AND ('postprandial glucose':ti,ab OR 'postprandial insulin':ti,ab OR 'glycemic control':ti,ab OR 'glycaemic control':ti,ab OR 'postprandial glycemia':ti,ab OR 'postprandial glycaemia':ti,ab OR 'insulin sensitivity':ti,ab OR 'c peptide':ti,ab OR 'flow-mediated dilation':ti,ab OR 'endothelial function':ti,ab OR 'vascular function':ti,ab OR 'endothelin-1':ti,ab).

Cochrane Library (CENTRAL)

Search: ("exercise snack*" OR "snack* exercise" OR "activity break*" OR "breaking sitting" OR "interrupting sedentary" OR "intermittent walk*" OR "brief bout*" OR "short bout*" OR "frequent bout*" OR "stair climb*" OR "resistance activit*" OR "high-intensity interval*" OR "exercise break*"):ti,ab AND ("randomized crossover" OR "randomised crossover" OR "acute crossover" OR "cross-over trial"):ti,ab AND ("postprandial glucose" OR "postprandial insulin" OR "glycemic control" OR "glycaemic control" OR "postprandial glycemia" OR "postprandial glycaemia" OR "insulin sensitivity" OR "C-peptide" OR "flow-mediated dilation" OR "endothelial function" OR "vascular function" OR "endothelin-1"):ti,ab.

Web of Science (Core Collection)

Search: TS=(("exercise snack*" OR "snack* exercise" OR "activity break*" OR "breaking sitting" OR "interrupting sedentary" OR "intermittent walk*" OR "brief bout*" OR "short bout*" OR "frequent bout*" OR "stair climb*" OR "resistance activit*" OR "high-intensity interval*" OR "exercise break*") AND ("randomized crossover" OR "randomised crossover" OR "acute crossover" OR "cross-over trial") AND ("postprandial glucose" OR "postprandial insulin" OR "glycemic control" OR "glycaemic control" OR "postprandial glycemia" OR "postprandial glycaemia" OR "insulin sensitivity" OR "C-peptide" OR "flow-mediated dilation" OR "endothelial function" OR "vascular function" OR "endothelin-1")).

CINAHL (EBSCOhost)

Search: ( TI("exercise snack*" OR "snack* exercise" OR "activity break*" OR "breaking sitting" OR "interrupting sedentary" OR "intermittent walk*" OR "brief bout*" OR "short bout*" OR "frequent bout*" OR "stair climb*" OR "resistance activit*" OR "high-intensity interval*" OR "exercise break*") OR AB("exercise snack*" OR "snack* exercise" OR "activity break*" OR "breaking sitting" OR "interrupting sedentary" OR "intermittent walk*" OR "brief bout*" OR "short bout*" OR "frequent bout*" OR "stair climb*" OR "resistance activit*" OR "high-intensity interval*" OR "exercise break*") ) AND ( TI("randomized crossover" OR "randomised crossover" OR "acute crossover" OR "cross-over trial") OR AB("randomized crossover" OR "randomised crossover" OR "acute crossover" OR "cross-over trial") ) AND ( TI("postprandial glucose" OR "postprandial insulin" OR "glycemic control" OR "glycaemic control" OR "postprandial glycemia" OR "postprandial glycaemia" OR "insulin sensitivity" OR "C-peptide" OR "flow-mediated dilation" OR "endothelial function" OR "vascular function" OR "endothelin-1") OR AB("postprandial glucose" OR "postprandial insulin" OR "glycemic control" OR "glycaemic control" OR "postprandial glycemia" OR "postprandial glycaemia" OR "insulin sensitivity" OR "C-peptide" OR "flow-mediated dilation" OR "endothelial function" OR "vascular function" OR "endothelin-1") ).

SportDiscus (EBSCOhost)

Search: ( TI("exercise snack*" OR "snack* exercise" OR "activity break*" OR "breaking sitting" OR "interrupting sedentary" OR "intermittent walk*" OR "brief bout*" OR "short bout*" OR "frequent bout*" OR "stair climb*" OR "resistance activit*" OR "high-intensity interval*" OR "exercise break*") OR AB("exercise snack*" OR "snack* exercise" OR "activity break*" OR "breaking sitting" OR "interrupting sedentary" OR "intermittent walk*" OR "brief bout*" OR "short bout*" OR "frequent bout*" OR "stair climb*" OR "resistance activit*" OR "high-intensity interval*" OR "exercise break*") ) AND ( TI("randomized crossover" OR "randomised crossover" OR "acute crossover" OR "cross-over trial") OR AB("randomized crossover" OR "randomised crossover" OR "acute crossover" OR "cross-over trial") ) AND ( TI("postprandial glucose" OR "postprandial insulin" OR "glycemic control" OR "glycaemic control" OR "postprandial glycemia" OR "postprandial glycaemia" OR "insulin sensitivity" OR "C-peptide" OR "flow-mediated dilation" OR "endothelial function" OR "vascular function" OR "endothelin-1") OR AB("postprandial glucose" OR "postprandial insulin" OR "glycemic control" OR "glycaemic control" OR "postprandial glycemia" OR "postprandial glycaemia" OR "insulin sensitivity" OR "C-peptide" OR "flow-mediated dilation" OR "endothelial function" OR "vascular function" OR "endothelin-1") ).
