# Supplementary material for: Acute effects of “exercise snacks” on postprandial glucose and insulin responses in populations at metabolic risk: a meta-analysis with exploratory meta-regression and vascular outcomes
Source: Front Endocrinol (Lausanne). 2026 Jul 22;17:1912963. doi: 10.3389/fendo.2026.1912963 (PMC13437454; doi:10.3389/fendo.2026.1912963)
Supplement: Supplementary file 2 [file SupplementaryFile2.docx]

**Supplementary Material**

This supplementary material provides additional details for outcomes that could not be subjected to formal meta‑analysis, as described in Section 3.7 of the main manuscript.

**C‑peptide iAUC**

After excluding the paediatric study (Broadney 2018) that did not meet the inclusion criteria for adult participants, only one study (Dempsey et al., 2016) reported C‑peptide incremental area under the curve (iAUC). That single study showed a statistically significant decrease in C‑peptide iAUC in the exercise‑snack condition compared with prolonged sitting (MD = −4.14 nmol·h/L, 95% CI −6.29 to −1.99, P < 0.001). No formal meta‑analysis was performed.

**Plasma Endothelin‑1 (ET‑1)**

A single study (Climie et al., 2018) reported that the total area under the curve for plasma endothelin‑1 over 5 hours was significantly lower in the simple‑resistance‑activity condition than in the prolonged‑sitting condition (MD = −1.1 pg·h/mL, P = 0.006). This finding is preliminary and based on one study only.

**Matsuda Insulin Sensitivity Index**

One study (Broadney et al., 2018) reported a significant improvement in the Matsuda insulin sensitivity index in the walking‑break condition compared with uninterrupted sitting. However, this study was excluded from the meta‑analysis because its participants were children and therefore did not meet the adult‑only eligibility criterion. Consequently, this result is not included in the main analysis.
