# Supplementary material for: Acute effects of “exercise snacks” on postprandial glucose and insulin responses in populations at metabolic risk: a meta-analysis with exploratory meta-regression and vascular outcomes
Source: Front Endocrinol (Lausanne). 2026 Jul 22;17:1912963. doi: 10.3389/fendo.2026.1912963 (PMC13437454; doi:10.3389/fendo.2026.1912963)
Supplement: Supplementary file 3 [file SupplementaryFile3.docx]

**Supplementary File: Reproducible R Code for the Meta‑Analysis**

\# =========================================================================

\# Final Reproducible R Code for:

\# "Acute Effects of Exercise Snacks on Postprandial Glucose and Insulin

\# Responses in Populations at Metabolic Risk"

\#

\# This script performs all primary and subgroup meta‑analyses, meta‑regression,

\# and generates publication‑quality forest plots.

\# All output numbers correspond to Table 3, Table 4, and the text.

\# =========================================================================

\# ---------- 0. Load required package ----------

\# If not installed, first run: install.packages("metafor")

library(metafor)

\# ---------- 1. Data: fully extracted effect sizes (n = 15 from 8 studies) ----------

dat <- data.frame(

Study_ID = c(

"Babir_2026","Chen_2018","Chen_2018",

"Climie_2018","Climie_2018",

"Dempsey_2016","Dempsey_2016","Dempsey_2016",

"Dempsey_2017",

"Francois_2014",

"Homer_2021","Homer_2021","Homer_2021",

"Larsen_2015","Larsen_2015"

),

Outcome_Category = c(

"Postprandial glucose",

"Postprandial glucose","Postprandial insulin",

"Vascular function","Vascular function",

"Postprandial glucose","Postprandial insulin","Postprandial insulin",

"Postprandial glucose",

"Postprandial glucose",

"Postprandial glucose","Postprandial insulin","Vascular function",

"Postprandial glucose","Postprandial insulin"

),

Specific_Measure = c(

"iAUC dinner",

"Glucose iAUC","Insulin iAUC",

"Femoral FMD (5-h mean)","ET-1 total AUC",

"Glucose iAUC","Insulin iAUC","C-peptide iAUC",

"Mean 22-h CGM glucose",

"Mean 3-h post-breakfast glucose",

"Glucose iAUCnet","Insulin iAUCnet","Femoral artery FMD (7-h mean)",

"Glucose iAUC","Insulin iAUC"

),

MD = c(

-19, -5.63, -2400,

3.1, -1.1,

-9.4, -1189, -4.137,

-2.7,

-1.4,

-4.4, -182, 0.4,

-1.3, -151

),

SE_MD = c(

6.76, 1.208, 476.7,

0.79, 0.367,

2.49, 315.4, 1.097,

0.72,

0.5,

2.02, 73.9, 0.19,

0.5, 73

),

Metabolic_Status = c(

"T2D non-insulin-treated",

"Central adiposity, normoglycemic","Central adiposity, normoglycemic",

"Overweight/obese, sedentary","Overweight/obese, sedentary",

"T2D (diet/metformin)","T2D (diet/metformin)","T2D (diet/metformin)",

"T2D (diet/metformin)",

"Insulin resistance (2 with T2D)",

"T2D (medication-controlled)","T2D (medication-controlled)","T2D (medication-controlled)",

"Overweight/obese, sedentary, non-diabetic","Overweight/obese, sedentary, non-diabetic"

)

)

\# ---------- 2. Glucose iAUC primary analysis (strictly k = 5) ----------

dat_glu <- subset(dat, Outcome_Category == "Postprandial glucose" &

grepl("iAUC", Specific_Measure))

cat("\n================ Glucose iAUC Primary Analysis (k = 5) ================\n")

res_glu <- rma(yi = MD, sei = SE_MD, data = dat_glu, method = "REML",

slab = Study_ID)

print(summary(res_glu))

pred_glu <- predict(res_glu)

cat(sprintf("95%% Prediction Interval: %.2f to %.2f\n\n", pred_glu$pi.lb, pred_glu$pi.ub))

\# ---------- 3. Dichotomise baseline metabolic status for meta‑regression ----------

dat_glu$Metabolic_Cat <- ifelse(

grepl("T2D|Insulin resistance", dat_glu$Metabolic_Status),

"Diabetic_IR",

"Normoglycemic_Obese"

)

\# ---------- 4. Meta‑regression (common‑τ² mixed‑effects model) ----------

cat("\n================ Meta‑Regression: Baseline Metabolic Status ================\n")

res_reg1 <- rma(yi = MD, sei = SE_MD, mods = ~ Metabolic_Cat,

data = dat_glu, method = "REML")

print(summary(res_reg1))

\# Extract subgroup marginal means (used in Table 4 and text)

cat("\n--- Subgroup Marginal Means (95% CI) ---\n")

pred_Diabetic <- predict(res_reg1, newmods = c(0))	# Diabetic_IR is reference

pred_Normo	<- predict(res_reg1, newmods = c(1))

cat(sprintf("Diabetic_IR: MD = %.2f, 95%% CI [%.2f, %.2f]\n",

pred_Diabetic$pred, pred_Diabetic$ci.lb, pred_Diabetic$ci.ub))

cat(sprintf("Normoglycemic_Obese: MD = %.2f, 95%% CI [%.2f, %.2f]\n\n",

pred_Normo$pred, pred_Normo$ci.lb, pred_Normo$ci.ub))

\# ---------- 5. Insulin iAUC primary analysis (k = 4) ----------

cat("\n================ Insulin iAUC Primary Analysis (k = 4) ================\n")

dat_ins <- subset(dat, Outcome_Category == "Postprandial insulin" &

grepl("Insulin iAUC", Specific_Measure))

res_ins <- rma(yi = MD, sei = SE_MD, data = dat_ins, method = "REML",

slab = Study_ID)

print(summary(res_ins))

\# ---------- 6. FMD: descriptive only (no formal meta‑analysis) ----------

cat("\n================ FMD (k = 2, descriptive) ================\n")

dat_fmd <- subset(dat, grepl("FMD", Specific_Measure))

for (i in 1:nrow(dat_fmd)) {

cat(sprintf("%s: MD = %.2f, 95%% CI [%.2f, %.2f]\n",

dat_fmd$Study_ID[i], dat_fmd$MD[i],

dat_fmd$MD[i] - 1.96 * dat_fmd$SE_MD[i],

dat_fmd$MD[i] + 1.96 * dat_fmd$SE_MD[i]))

}

\# =========================================================================

\# 7. Publication‑ready forest plots

\# =========================================================================

\# --- Figure 3: Glucose forest plot (with weights and prediction interval) ---

png("Figure_3_Glucose.png", width = 3600, height = 2400, res = 300)

par(mar = c(6, 8, 4, 6))

forest(res_glu, showweights = TRUE, pi = TRUE, slab = dat_glu$Study_ID,

main = "Effects of Exercise Snacks on Glucose iAUC",

xlab = "Mean Difference (mmol·h/L)",

xlim = c(-50, 20), ylim = c(-4, 8), cex = 1.1)

dev.off()

\# --- Figure 5: Insulin forest plot ---

png("Figure_5_Insulin.png", width = 3600, height = 2400, res = 300)

par(mar = c(6, 8, 4, 6))

forest(res_ins, showweights = TRUE, pi = TRUE, slab = dat_ins$Study_ID,

main = "Effects of Exercise Snacks on Insulin iAUC",

xlab = "Mean Difference (pmol·h/L)",

xlim = c(-8000, 5500), alim = c(-4000, 1000),

ylim = c(-4, 7), cex = 1.1)

dev.off()

\# --- Figure 4: Subgroup forest plot (replaces bubble plot) ---

\# Order studies by subgroup

dat_glu_sub <- dat_glu[order(dat_glu$Metabolic_Cat), ]

k_t2d	<- sum(dat_glu_sub$Metabolic_Cat == "Diabetic_IR")

k_normo <- sum(dat_glu_sub$Metabolic_Cat == "Normoglycemic_Obese")

\# Define custom rows so that subgroups are visually separated

my_rows <- c((k_normo + 4):(k_normo + 3 + k_t2d), 1:k_normo)

\# Run intra‑subgroup models (for display only, p‑values not reported separately)

res_t2d	<- rma(yi = MD, sei = SE_MD,

data = dat_glu_sub[dat_glu_sub$Metabolic_Cat == "Diabetic_IR", ])

res_normo <- rma(yi = MD, sei = SE_MD,

data = dat_glu_sub[dat_glu_sub$Metabolic_Cat == "Normoglycemic_Obese", ])

png("Figure_4_Subgroup.png", width = 3600, height = 2800, res = 300)

par(mar = c(6, 8, 4, 2))

forest(dat_glu_sub$MD, sei = dat_glu_sub$SE_MD,

rows = my_rows, slab = dat_glu_sub$Study_ID,

xlab = "Mean Difference (mmol·h/L)",

main = "Subgroup Analysis by Baseline Metabolic Status",

xlim = c(-45, 25), ylim = c(-3.5, 12), cex = 1.1)

addpoly(res_t2d, row = (k_normo + 2.5), cex = 1.1, mlab = "Summary: T2D/IR Group")

addpoly(res_normo, row = -1, cex = 1.1, mlab = "Summary: Normoglycemic/Obese")

text(-45, 10.5, pos = 4, "Diabetic / Insulin Resistant", font = 2, cex = 1.2)

text(-45, k_normo + 1.5, pos = 4, "Normoglycemic / Obese", font = 2, cex = 1.2)

dev.off()

cat("\n===== Analyses complete. Figures saved as PNG. =====\n")
